# Laser-modified titanium surfaces induce sex-dimorphic secretion of angiogenic factors by gingiva-derived mesenchymal stromal cells

**DOI:** 10.1016/j.jds.2025.09.013

**Published:** 2025-11-03

**Authors:** Ting-Han Chang, Hsin-Han Hou, Yi-Shing Shieh, Her-Hsiung Huang, Da-Yo Yuh

**Affiliations:** a Institute of Oral Biology, College of Dentistry, National Yang Ming Chiao Tung University, Taipei, Taiwan; b Department of Dentistry, College of Dentistry, National Yang Ming Chiao Tung University, Taipei, Taiwan; c Department of Dentistry, National Taiwan University Hospital, Taipei, Taiwan; d Graduate Institute of Oral Biology, School of Dentistry, National Taiwan University, Taipei, Taiwan; e Department of Dentistry, Tri-Service General Hospital, Taipei, Taiwan; f School of Dentistry, College of Oral Medicine, National Defense Medical University, Taipei, Taiwan; g Department and Graduate Institute of Biochemistry, National Defense Medical University, Taipei, Taiwan; h Institute of Oral Tissue Engineering and Biomaterials, College of Dentistry, National Yang Ming Chiao Tung University, Taipei, Taiwan; i Faculty of Dentistry, Chulalongkorn University, Bangkok, Thailand

**Keywords:** Mesenchymal stromal cells, CCN1, EDIL3, Extracellular vesicles, Titanium, Laser

## Abstract

**Background/purpose:**

Dental implants have been applied to restore missing dentition for more than fifty years. However, whether different surface modification on implant provide topographical cues that alter sex-specific effect on peri-implant angiogenesis remains unclear. This study aimed to investigate whether titanium surface modifications influence sex-specific chromatin accessibility, gene expression, and angiogenesis-related functions in human gingiva-derived mesenchymal stromal cells (GMSCs).

**Materials and methods:**

We collected health gingival tissues from sixteen subjects (eight females and eight males) and isolated primary GMSCs. The primary GMSCs were cultured on three types of titanium surfaces: machined, lasered, and SLA (sand-blasted, large-grit, acid-etched). Chromatin accessibility and gene expression were assessed via ATAC-seq and RNA-seq, respectively, with further validation through qRT-PCR, ELISA, and western blot. Functional angiogenic potential was evaluated using both direct and indirect tube formation assays.

**Results:**

Our findings revealed that titanium surface modifications induce sex-dimorphic effects on the secretion of angiogenic factors CCN1 and EDIL3. Laser-modified titanium significantly increased the secretion of extracellular vesicles enriched with both CCN1 and EDIL3 compared to machined surfaces. Interestingly, female GMSCs secreted higher levels of EDIL3, while male GMSCs secreted more CCN1 on lasered titanium surfaces. Moreover, lasered titanium demonstrated superior angiogenic potential in both direct and indirect tube formation assays compared with machined and SLA titanium.

**Conclusion:**

This study suggests that laser-modified microchannels on titanium surfaces provide bioactive topographical cues that enhance the secretion of extracellular vesicles enriched with angiogenic factors—particularly CCN1 and EDIL3—by GMSCs, which may play key roles in promoting angiogenesis and supporting early-stage osseointegration.

## Introduction

Dental implants have been successfully applied to restored the lost teeth for over fifty years.^1^ Unlike orthopedic implants placed beneath periosteum, dental implants penetrate periosteum and oral mucosa, exposing them to immune challenges from oral microbiome.^2^ The supra-crestal periodontal tissue serves as a unique biological seal around natural teeth, maintaining osteoimmune homeostasis.^3^^,^^4^ However, stable transmucosal barriers around implants are hard to achieve due to poor vascularization at titanium surfaces.^5^^,^^6^ Periodontal stromal cells harbor neural crest-like progenitors that respond to mechanical and topographical cues, regulating immune responses and supporting tissue integration.^7^^,^^8^ Laser-modified titanium surfaces have been shown to promote perpendicular collagen fiber insertion at supra-crestal attachments and reduce peri-implant bone loss.^9^^,^^10^ However, whether laser-modified microchannels provide topographical cues that alter sex-specific chromatin accessibility and gene expression to enhance angiogenesis—an essential step in early osseointegration—remains unclear.

Emerging evidence suggests that biological sex affects cellular responses to titanium, potentially impacting implant osseointegration and long-term outcomes.^11^ Female osteoblasts show greater sensitivity to estrogen and surface microtopography, while male osteoblasts respond more to vitamin D.^11^ Clinically, implants in males show higher failure rates and greater marginal bone loss with male sex identified as a risk factor for non-osseointegration.^12^^,^^13^ While gingiva-derived mesenchymal stromal cells (GMSCs) play a pivotal role in peri-implant tissue sealing and immune regulation,^8^ the sex-specific responses of GMSCs to different titanium surface modifications remain largely unexplored.

This study investigated whether laser-modified titanium surfaces altered chromatin accessibility and gene expression in primary human GMSCs and affected angiogenesis. We performed assay for transposase-accessible chromatin (ATAC)-sequencing and RNA-sequencing on male and female donor GMSCs cultured on machined, laser-modified, and SLA titanium surfaces. Laser modification induced sex-specific differences in extracellular vesicles (EVs) secretion enriched with angiogenic factors—especially CCN1 and EDIL3—showing higher expression than machined surfaces. Using direct and indirect tube formation assays, we demonstrated that laser-modified surfaces promote greater angiogenesis than machined or SLA-treated surfaces. These results suggest laser-modified microchannels on titanium stimulate GMSCs to secrete pro-angiogenic factors, potentially enhancing early vascularization.

## Materials and methods

### Tissue collection

This study was conducted in accordance with human subject research guidelines and was approved by the Institutional Review Board of Tri-Service General Hospital (C202505047). Gingival tissues were collected from sixteen participants, eight females (59.6 ± 9.7 years) and eight males (52.5 ± 9.1 years), during second-stage implant surgery (Table 1).

**Table 1:** Age and sex of human donors (n = 16) used for gingiva-derived mesenchymal stromal cells (GMSCs) isolation and subsequent experiments.

Sex	Experiment	Age	Average ± SD
Female	qRT-PCR, ELISA, western blot, NTA, tube formation	46	59.6 ± 9.7
		50 63	
		67	
		72	
	Organoid	50	
		57	
		72	
Male	qRT-PCR, ELISA, western blot, NTA, tube formation	42	52.5 ± 9.1
		50 51	
		59	
		69	
	Organoid	41	
		47	
		61	

Abbreviations: SD = Standard deviation; qRT-PCR = Quantitative real-time polymerase chain reaction; ELISA = Enzyme-linked immunosorbent assay; NTA = nanoparticle tracking analysis.

### Isolation of gingiva-derived mesenchymal stromal cells

**Figure 1:**
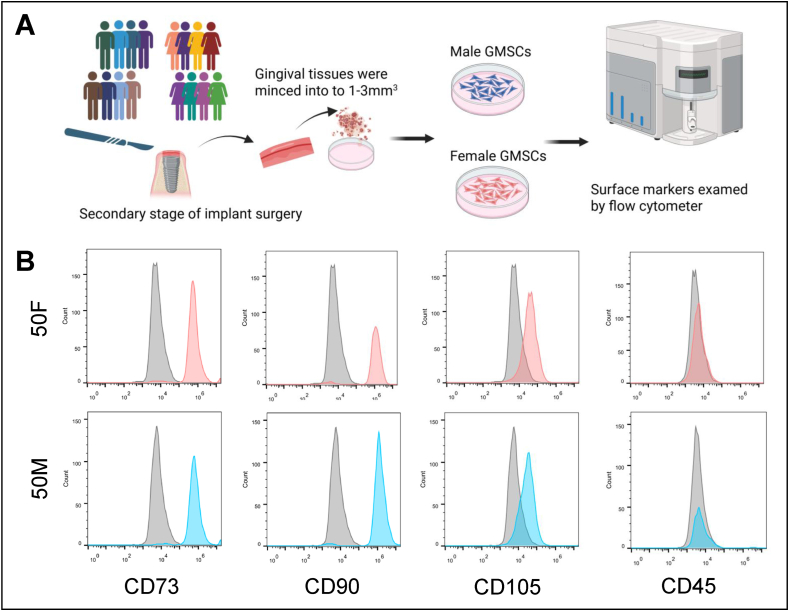
Isolation and characterization of primary human gingiva-derived mesenchymal stromal cells (GMSCs). (A) Gingival tissues were collected from eight males and eight females during second stage of implant surgery. (B) Flow cytometry analysis confirmed that GMSCs from both genders expressed cluster of differentiation (CD)73, CD90, and CD105, and were negative for the hematopoietic marker CD45. 50M, 50-year-old male. 50F, 50-year-old female.

Primary GMSCs were isolated according to previously established protocols.^14^ Briefly, fresh human gingival tissues (at least 3–6 mm^3^) were minced into 1–3 mm^3^ fragments and subjected to enzymatic digestion using 0.2 % collagenase I for 2 h at 37 °C. Then the dissociated cells were cultured in α-minimum essential medium (α-MEM: Invitrogen, Carlsbad, CA, USA) supplemented with 10 % fetal bovine serum (FBS: Zen-Bio, Inc., Durham, NC, USA) and 1 % penicillin/streptomycin. Flow cytometry was performed to confirm mesenchymal stromal cell surface markers (Fig. 1). Cells under passage six were employed in subsequent experiments.

### Experiments on titanium discs

**Figure 2:**
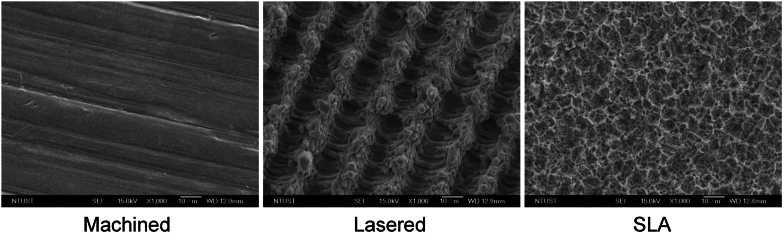
Titanium surface modifications. Representative images of the three titanium surface modifications used in the study: machined, lasered, and SLA (sand-blasted, large-grit, acid-etched) discs, acquired under scanning electron microscopy (SEM) in backscattered electron mode at 1000× magnification.

Titanium discs (diameter: 15 mm; thickness: 2 mm) were made from bars of commercially pure, grade IV titanium and prepared with three surface modifications: machined, lasered, and SLA by Biomate (Taipei, Taiwan) (Fig. 2).^15^ Briefly, SLA surfaces were created by sandblasting machined titanium discs with 250–500 μm Al_2_O_3_ particles, followed by HCl/H_2_SO_4_ acid etching. Lasered surfaces were produced by ablating machined discs using a Q-switched DPSS Nd:YVO_4_ laser (335 nm, 1 W, 50 mm/s), scoring parallel lines under 1 atm. Discs were placed in 24-well plates, rinsed with culture medium, and seeded with primary GMSCs (5 × 10^5^ cells/disc) in α-MEM containing 10 % exosome-depleted FBS and 1 % penicillin–streptomycin (2 ml/well). After 72 h, conditioned media were collected for ELISA and tube formation assays, while cells were harvested for sequencing, qRT-PCR, and western blot analysis.

### Genomic sequencing

GMSCs were cultured on titanium discs (four discs/group; one 50-year-old female and one 50-year-old male donor). After 72 h, cells were dissociated; half were resuspended in PBS for ATAC-seq, and the remainder in TRIzol (15596018, Invitrogen) for RNA-seq. Library preparation was performed at the Instrument Center of the National Defense Medical Center, and sequencing was conducted by Genomics BioSci & Tech Co., Ltd. (New Taipei City, Taiwan). ATAC-seq data were visualized using Integrative Genomics Viewer (IGV) and analyzed by SeqMonk. RNA-seq data were analyzed using CLC Genomics Workbench (QIAGEN, Hilden, Germany). Differentially expressed genes were defined by fold change>1.3 and *P* < 0.05 and visualized using Venn diagrams. Volcano plots were created with the Enhanced Volcano R package using a *P*-value cutoff of 0.3 and log_2_ fold change>0.5. Gene Ontology (GO) enrichment analysis compared gene expression between laser-treated and machined surfaces, with *P* < 0.05 considered significant.

### Quantitative real-time PCR (qRT-PCR)

RNA was extracted from GMSCs using the GENEzol kit (GZR100, Geneaid, New Taipei City, Taiwan) and reverse-transcribed with the Power SYBR Green RNA-to-CT 1-Step Kit (4389986, Applied Biosystems, Thermo Fisher Scientific, Waltham, MA, USA). qRT-PCR was performed on a Bio-Rad CFX96 system. Gene expression was normalized to RPS18 and calculated using the 2^−ΔΔCt^ method. Primer sequences are listed in Table 2.

**Table 2:** Sequences of primers used for qRT-PCR.

Gene	Primer sequences (5′-3′)
CCN1	Forward: CTCGCCTTAGTCGTCACCC
	Reverse: CGCCGAAGTTGCATTCCAG
ADM2	Forward: TACACGCAGTGCTGGTACG
	Reverse: CTGCTCGTCCAGACATGGC
EDIL3	Forward:GCGAATGGAACTTCTTGGCTGTG
	Reverse: GAGCGTTCTGAAGATGCTGGAG
β-actin	Forward: CACCATTGGCAATGAGCGGTTC
	Reverse: AGGTCTTTGCGGATGTCCACGT
RPS18	Forward: GCAGAATCCACGCCAGTACAAG
	Reverse: GCTTGTTGTCCAGACCATTGGC

Abbreviations: CCN1 = Cellular communication network factor 1; ADM2 = Adrenomedullin 2; EDIL3 = EGF like repeats and discoidin domains 3; RPS18 = Ribosomal protein S18.

### Enzyme-linked immunosorbent assay (ELISA)

Conditioned medium was centrifuged at 300×*g* for 10 min at 4 °C, and the supernatant was subsequently treated with or without 0.1%Triton X. EDIL3 and CCN1 levels were then quantified using the Human EDIL3 DuoSet ELISA (DY6046–05, R&D systems, Abingdon, UK) and the CCN1 ELISA (DCYR10, R&D systems).

### Western blot study

**Figure 3:**
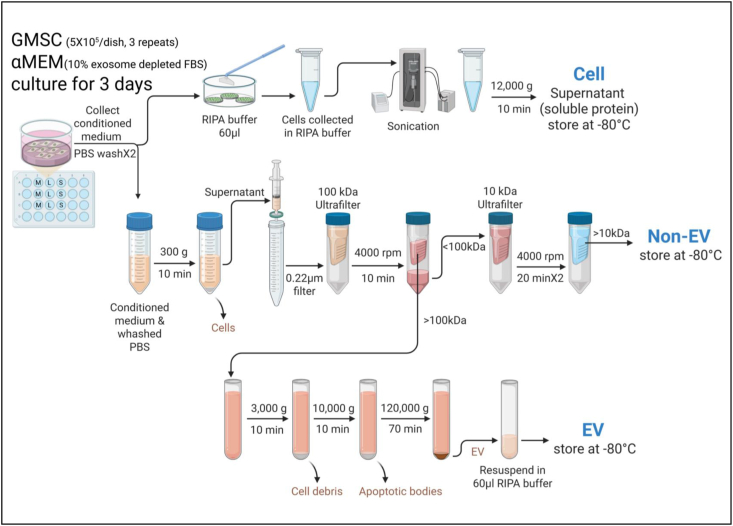
Schematic of protocol for isolating extracellular vesicle (EV) and non-EV components. Created in https://BioRender.com. For cells: cells were lysed directly on titanium discs using 60 μl RIPA buffer (with protease and phosphatase inhibitors), followed by sonication and centrifugation (12,000×*g*, 10 min, 4 °C). Supernatants were stored at −20 °C. For non-EV: conditioned medium was centrifuged (300×*g*, 10 min), filtered (0.22 μm), and ultrafiltered (<100 kDa) by 100 kDa and 10 kDa cutoffs. The final flow-through (10–100 kDa) was concentrated, centrifuged (12,000×*g*, 10 min), and stored. For EV: Retentates (>100 kDa) were centrifuged (3000×*g*, then 10,000×*g*), followed by ultracentrifugation (120,000×*g*, 70 min, 4 °C). EV pellets were resuspended in 60 μl RIPA buffer and stored. GMSCs, human gingiva-derived mesenchymal stromal cells. α-MEM, α-minimum essential medium. FBS, fetal bovine serum. PBS, phosphate-buffered saline. M, machined. L, lasered. S, SLA (sand-blasted, large-grit, acid-etched). RIPA, radioimmunoprecipitation assay.

The conditioned medium was separated into non-EV and EV fractions, and the corresponding cells were collected for western blot analysis (Fig. 3) as previously described protocol.^16^ Protein concentrations were determined using Pierce BCA Protein Assay Kits (23225, Thermo Fisher Scientific). Thirty micrograms of protein per sample were separated on 10 % SDS-polyacrylamide gels and transferred onto nitrocellulose membranes (BioRad, Hercules, CA, USA). Membranes were incubated overnight at 4 °C with primary antibodies (Table 3), followed by incubation with horseradish peroxidase-conjugated secondary antibodies 1 h. Protein bands were visualized using Clarity Max Western ECL Substrate (1705062, BioRad) and the touch imager (e-Blot, Shanghai, China).

**Table 3:** Antibodies used for western blotting.

Antibody	Species	Dilution	Company (Catalog number)
CCN1	Rabbit	1:1000	Cell Signaling Technology (14479)
EDIL3	Rabbit	1:1000	Abcam (ab190692)
ADM2	Rabbit	1:1000	Invitrogen (PA5-72030)
TSG101	Rabbit	1:1000	Cell Signaling Technology (72312)
Flotillin-1	Rabbit	1:1000	Cell Signaling Technology (18634)
β-actin	Mouse	1:1000	Santa Cruz (sc-47778)
Anti-rabbit IgG-HRP	Goat	1:5000	Santa Cruz (sc-2004)
Anti-mouse IgG-HRP	Goat	1:5000	Santa Cruz (sc-2005)

Abbreviations: CCN1 = Cellular communication network factor 1; EDIL3 = EGF like repeats and discoidin domains 3; ADM2 = Adrenomedullin 2; TSG101 = Tumor susceptibility gene 101; IgG = Immunoglobulin G; HRP = Horseradish peroxidase.

#### Tube formation assays

Human Umbilical Vein Endothelial Cells (HUVEC) were purchased from ScienCell Research Laboratories (Carlsbad, CA, USA) and cultured in ECM medium (ScienCell) with 10 % FBS, and passages below 10 were used for tube formation assays. HUVECs (2 × 10^4^ in 75 μl ECM) were labeled with CellTracker Red (C34552, Invitrogen) and seeded onto Matrigel-coated wells (n = 3/group). In indirect tube formation assays, GMSC-conditioned medium (75 μl/well) from titanium discs was added, and HUVECs were cultured on Matrigel with reduced growth factors for 72 h. Tube-like structures were imaged with live-cell fluorescent microscopy (ImageXpress Pico, Molecular Devices) and quantified using ImageJ.^17^

To further evaluate the effect of direct GMSC-to-HUVEC interaction on angiogenesis around implant surface, titanium rods (2.3 mm × 5.3 mm) with a 1 mm laser-treated base or SLA-treated base were defined as the laser-modified group or SLA-modified group, respectively. GMSCs (2 × 10^5^), labeled with CellTracker Green (C7025, Invitrogen) and Hoechst 33342 (Immunochemistry Technologies, Bloomington, MN, USA), were mixed in 200 μL of gelatin–methacrylamide (GelMA, Cellink, San Diego, CA, USA) (α-MEM: GelMA = 1:3) with no growth factors and loaded into 8-well chamber slides. Rods with machined, laser, or SLA surfaces were inserted, crosslinked under 405 nm light (2 min), and incubated in α-MEM:ECM (1:1) for 72 h. HUVECs (2 × 10^5^), labeled with CellTracker Red and Hoechst, were then seeded onto the GelMA and cultured for another 72 h. The direct interaction images between GMSCs and HUVEC on titanium rods were captured using the THUNDER Imager (Leica Camera, Wetzlar, Germany) and processed with Imaris software (Bitplane Inc., St. Paul, MN, USA). Captured images were converted to 8-bit grayscale and analyzed using ImageJ.^17^ The surface of the titanium rods was analyzed using scanning electron microscopy in backscattered electron mode.

### Transfection

After GMSCs were seeded on titanium discs overnight, knockdown experiments were performed using Lipofectamine RNAiMAX Transfection Reagent (13778075, Invitrogen) with 30 nM Silencer Select siRNAs for 24 h (negative control siRNA, 4390843; siCYR61, 4390824, ID: s7244; siEDIL3, 4392420, ID: s223075, Invitrogen) according to the manufacturer's instructions. Subsequently, the medium was replaced with fresh exosome-free α-MEM and cells were cultured for an additional 48 h. Conditioned medium was collected for the tube formation assay, and GMSCs were harvested for RNA extraction. mRNA expression was validated using TaqMan Fast Advanced Master Mix (4444556, Invitrogen) with TaqMan Gene Expression Assays (Invitrogen) for CYR61 (Hs00155479_m1), EDIL3 (Hs00964112_m1), and GAPDH (Hs02786624_g1), according to the manufacturer's protocol. HUVECs for tube formation assay were labeled with Calcein AM (C1430, Invitrogen) (2 × 10^4^ in 100 μl of conditioned medium) were seeded onto Matrigel-coated wells (n = 3/group).

### Statistical analysis

Data were evaluated by Student's t test for comparing two groups when appropriate. In cases of multiple groups, statistical analysis was performed through one-way ANOVA analysis with Tukey's multiple comparison test. All analyses were done using GraphPad Prism 10 (GraphPad Software, Boston, MA, USA). A value of *P* < 0.05 was considered statistically significant.

## Results

### Lasered titanium increased chromatin accessibility of CCN1 and EDIL3 in gingival stromal cells

**Figure 4:**
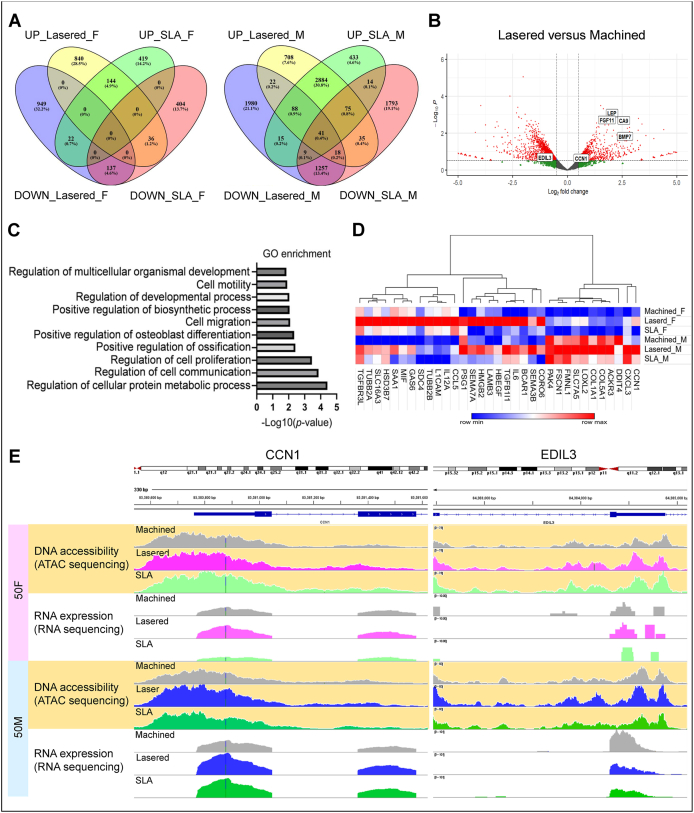
Laser-modified titanium upregulates angiogenesis-related genes in human gingiva-derived mesenchymal stromal cells (GMSCs). Assay for transposase-accessible chromatin sequencing (ATAC-seq) and RNA-sequencing (RNA-seq) were performed on GMSCs cultured on machined, lasered, and SLA (sand-blasted, large-grit, acid-etched) titanium discs for 72 h (n = 2 per group; age-matched 50-year-old female and male). (A) Venn diagrams show differentially upregulated (UP) and downregulated (DOWN) genes in the lasered group and SLA group compared to the machined group, respectively. (B) Volcano plot highlights differentially expressed angiogenesis-related genes. (C) Gene Ontology (GO) enrichment analysis of genes upregulated in the lasered group versus the machined group. (D) Thirty-three genes were identified at the intersection of GO:0016477 (cell migration) and genes upregulated in both female and male GMSCs on laser-modified versus machined surfaces. The heatmap shows shared migration-related genes upregulated in both sexes. (E) Chromatin accessibility and RNA expression levels of CCN1 and EDIL3. F, female. M, male. CCN1, cellular communication network factor 1. EDIL3, EGF like repeats and discoidin domains 3.

**Figure 5:**
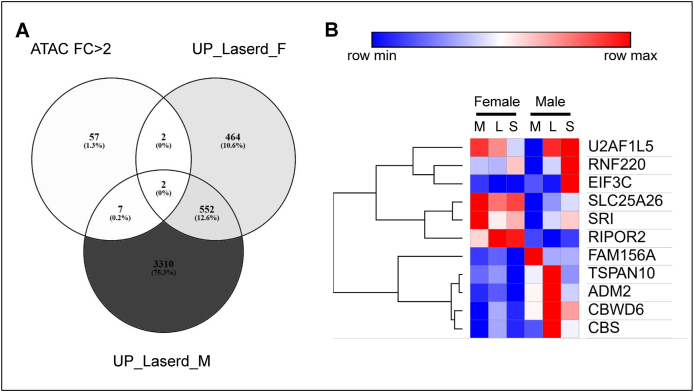
Integrated analysis of assay for transposase-accessible chromatin sequencing (ATAC-seq) and RNA-sequencing (RNA-seq) in primary human GMSCs cultured on titanium discs with different surface modifications. Primary human gingiva-derived mesenchymal stromal cells (GMSCs) from age-matched 50-year-old female and male donor were cultured on machined, laser-modified, and SLA (sand-blasted, large-grit, acid-etched) titanium discs for 72 h prior to ATAC-seq and RNA-seq analysis. (A) A total of 68 genes showed more than 2-fold changes in chromatin accessibility in the ATAC-seq data. The Venn diagram illustrates the overlap between genes with >2-fold change in ATAC-seq (ATAC FC > 2), genes upregulated in the female laser-modified group versus the female machined group (UP_Lasered_F), and genes upregulated in the male laser-modified group versus the male machined group (UP_Lasered_M). (B) A heatmap displays 11 genes at the intersection of ATAC FC > 2 with UP_Lasered_F and with UP_Lasered_M.

To assess how titanium surface modifications affect chromatin accessibility and gene expression in GMSCs, we cultured GMSCs from a 50-year-old female and male on machined, lasered, and SLA titanium discs for three days, followed by ATAC-seq and RNA-seq. Differential gene expression in lasered and SLA groups, relative to machined group, was analyzed separately by sex (Fig. 4A). Volcano plots showed upregulation of angiogenesis-related genes, including LEP, CA9, FGF11, BMP7, CCN1, and downregulation of EDIL3 in the lasered group (Fig. 4B). GO analysis showed that upregulated genes in lasered group were enriched in cell migration pathway (Fig. 4C). In cell migration-related genes, CCN1 was consistently upregulated in both sexes in the lasered group (Fig. 4D) and showed increased chromatin accessibility and RNA expression (Fig. 4E). Interestingly, while EDIL3 RNA expression was downregulated in the male lasered group, chromatin accessibility at EDIL3 locus was increased compared to machined group. Additionally, genes with >2-fold ATAC-seq changes were compared, identifying overlapping upregulated genes in both sexes (Fig. 5). Among these, ADM2 was notably upregulated in the lasered group. These findings suggest that laser-modified titanium enhances cell migration and chromatin accessibility of angiogenesis-related genes such as CCN1, EDIL3, and ADM2 in human GMSCs.

### Lasered titanium induced sex-dimorphic EDIL3 and CCN1 secretion in gingival stromal cells

**Figure 6:**
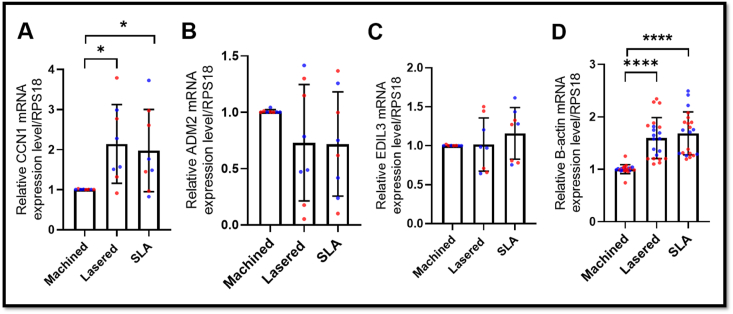
Laser-modified titanium surfaces upregulate CCN1 expression in human gingiva-derived mesenchymal stromal cells (GMSCs). Relative gene expression levels of ADM2, CCN1, EDIL3, and β-actin in GMSCs cultured on three different titanium disc surfaces: machined, lasered, and SLA (sand-blasted, large-grit, acid-etched). (A) CCN1 mRNA levels were significantly upregulated in the laser-treated and SLA groups compared to the machined group. (B) and (C) No significant differences in ADM2 and EDIL3 expression were observed among the three groups. (D) β-actin expression was significantly increased in the laser-treated and SLA groups compared to the machined group. Data are means ± standard deviation (n = 8 per group, 4 females and 4 males). Paired t-test. ∗*P* < 0.05, ∗∗∗∗*P* < 0.0001. mRNA, messenger ribonucleic acid. CCN1, cellular communication network factor 1. ADM2, adrenomedullin 2. EDIL3, EGF like repeats and discoidin domains 3. RPS18, ribosomal protein S18.

**Figure 7:**
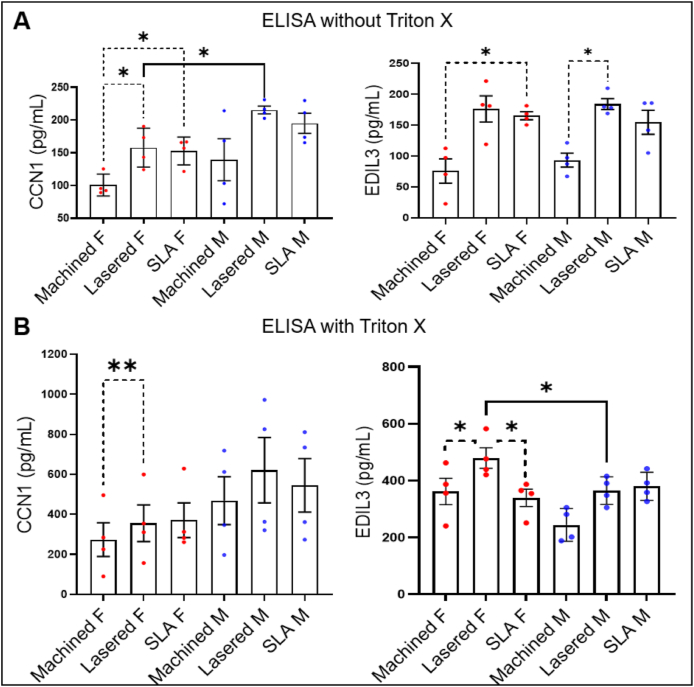
Sex-dimorphic secretion of EDIL3 and CCN1 by human gingiva-derived mesenchymal stromal cells (GMSCs). ELISA assays were performed to quantify CCN1 and EDIL3 levels in conditioned medium collected from GMSCs cultured on machined, lasered, and SLA (sand-blasted, large-grit, acid-etched) titanium discs for 72 h. (A) ELISA results from conditioned medium without Triton X treatment. Data are mean ± standard deviation (n = 8; four females and four males). Dotted line: paired t-test. Solid line: unpaired t-test. ∗*P* < 0.05. (B) ELISA results from conditioned medium treated with Triton X. Data are mean ± standard deviation (n = 8; four females and four males). Dotted line: paired t-test. Solid line: unpaired t-test. ∗*P* < 0.05, ∗∗*P* < 0.01. F, female. M, male. CCN1, cellular communication network factor 1. EDIL3, EGF like repeats and discoidin domains 3.

**Figure 8:**
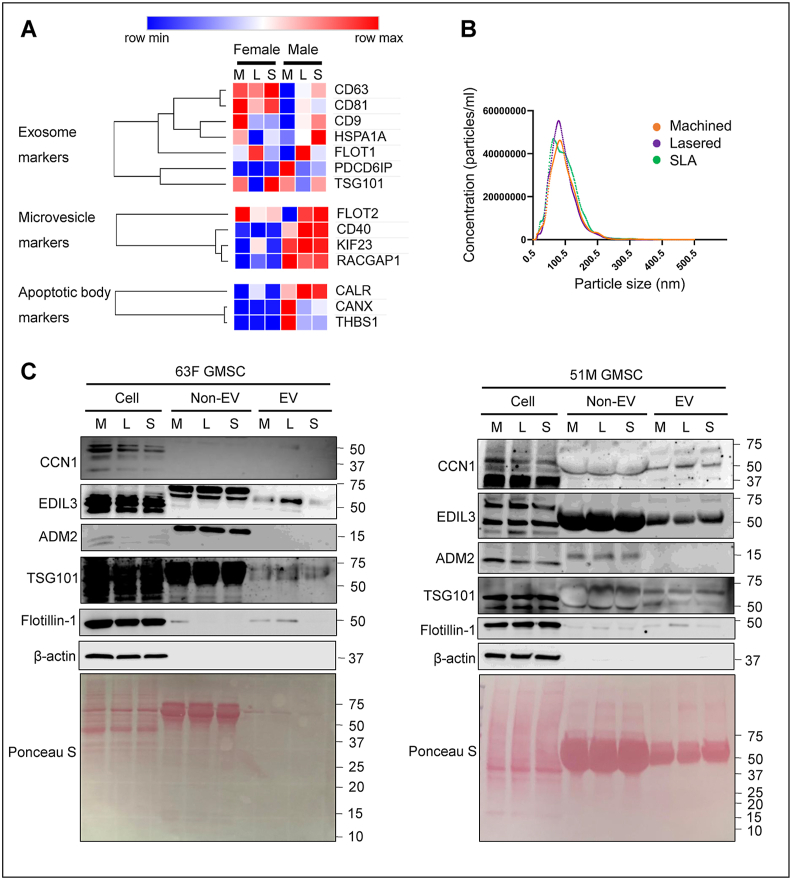
Laser-modified titanium promotes the secretion of extracellular vesicles enriched with CCN1 and EDIL3 from human gingiva-derived mesenchymal stromal cells (GMSCs). (A) Primary GMSCs from one female and one male donor were cultured on machined, lasered, and SLA discs for 72 h, followed by ATAC-seq and RNA-seq analysis. Heatmaps show exosome-related genes. M, machined. L, lasered. S, SLA (sand-blasted, large-grit, acid-etched). (B) Primary GMSCs were cultured on machined, laser-treated, and SLA (sand-blasted, large-grit, acid-etched) titanium discs for 72 h. The conditioned medium was collected and centrifuged at 300×*g* for 10 min. The supernatant was then filtered through a 0.22 μm membrane, and particle size was analyzed using nanoparticle tracking analysis (NTA). Data are presented as mean values (n = 8 per group; 4 females and 4 males). (C) Western blot analysis was performed to evaluate CCN1, EDIL3 and ADM2 protein levels in cells, extracellular vehicles (EVs), and non-extracellular vesicle (non-EV) fractions derived from GMSCs (51-year-old male, 51M, and 63-year-old female, 63F) were cultured on machined (M), lasered (L), and SLA (sand-blasted, large-grit, acid-etched, S) titanium discs for 72 h. Laser-modified surfaces increased CCN1 in male GMSC EVs and EDIL3 in female EVs compared to machined surfaces. Lower panels show Ponceau S staining of the corresponding PVDF membranes. Results shown are representative of at least three independent experiments. CCN1, cellular communication network factor 1. ADM2, adrenomedullin 2. EDIL3, EGF like repeats and discoidin domains 3. TSG101, tumor susceptibility gene 101.

To validate whether laser-modified titanium surfaces induce angiogenesis-related gene expression, we cultured primary GMSCs and found that CCN1 RNA was significantly upregulated in both lasered and SLA groups compared to machined (*P* < 0.05), consistent with the ATAC-seq and RNA-seq findings (Fig. 6). ADM2 and EDIL3 RNA levels showed no significant changes. β-actin RNA was markedly increased on lasered and SLA surfaces (*P* < 0.0001) (Fig. 6D). Furthermore, ELISA revealed higher CCN1 secretion by female GMSCs (*P* < 0.05) and increased EDIL3 secretion by male GMSCs (*P* < 0.05) on lasered titanium compared to the machined group (Fig. 7A). Triton X-100 treatment further elevated CCN1 and EDIL3 levels (Fig. 7B), indicating their encapsulation in extracellular vesicles. Interestingly, male GMSCs secreted higher levels of CCN1 (*P* < 0.05), while permeabilization revealed greater EDIL3 release from female GMSCs (*P* < 0.05) (Fig. 7). We then analyzed the expression of EV-related genes from RNA sequencing of GMSCs according to the previous publication, and lasered group show higher exosome markers flotillin-1 (Fig. 8A).^18^ We further characterized EV size and protein content in conditioned medium (Fig. 8B and C). Ultrafiltration and ultracentrifugation followed by western blot revealed higher flotillin-1 in EVs (>100 kDa) from GMSCs on lasered titanium in both sexes, with elevated CCN1 in male EVs and EDIL3 in female EVs (Fig. 8C). Taken together, our findings demonstrate that laser-modified titanium surfaces enhance the secretion of extracellular vesicles enriched with angiogenesis-related proteins, such as EDIL3 and CCN1, and that these effects exhibit sex-specific differences in human GMSCs.

### Lasered titanium promoted gingival stromal cell-induced angiogenesis in endothelial cells

**Figure 9:**
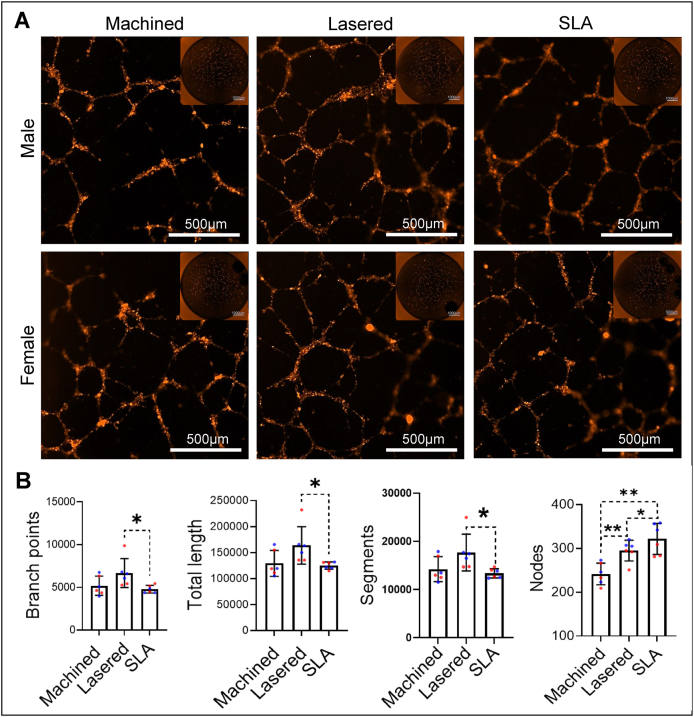
Lasered titanium promoted gingival stromal cell-induced angiogenesis in endothelial cells. A tube formation assay was conducted over 72 h using human umbilical vein endothelial cells (HUVECs) stimulated with conditioned medium from human gingiva-derived mesenchymal stromal cells (GMSCs) cultured on machined, lasered, and SLA (sand-blasted, large-grit, acid-etched) titanium discs. (A) Representative images of tube-like structures in each well were captured using the ImageXpress Pico system. (B) The tube formation was quantified using ImageJ (Fiji distribution). Data are mean ± standard deviation (n = 6, three females and three males). Paired t-test. ∗*P* < 0.05, ∗∗*P* < 0.01.

**Figure 10:**
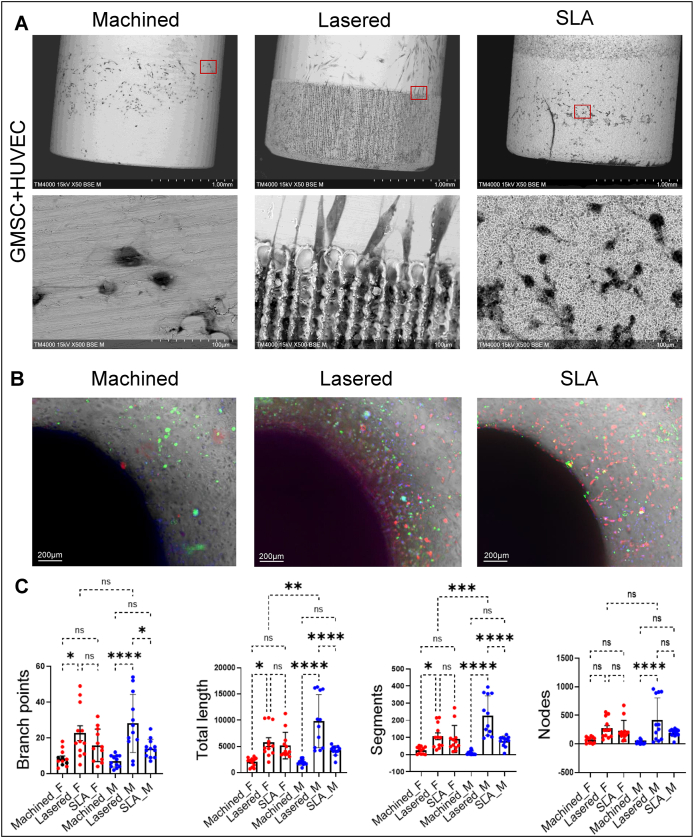
Interaction of HUVECs and GMSCs around titanium rods. Human gingiva-derived mesenchymal stromal cells (GMSCs) were mixed with gelatin–methacrylamide (GelMA), and the machined, lasered, and SLA (sand-blasted, large-grit, acid-etched) titanium rods were embedded into the GelMA-cell mixture. Human umbilical vein endothelial cells (HUVECs) were then seeded onto the GelMA surface to establish a 3D angiogenesis organoid model. (A) Scanning electron microscopy (SEM) in backscattered electron (BSE) mode was used to analyze cell–titanium interactions after three days of organoid culture, following the removal of titanium rods from the GelMA-cell mixture. Upper panels: 50 × magnification; lower panels: 500 × magnification of the red-marked region in the upper panels. (B) Tube formation around the titanium rods. Red: HUVECs. Green: GMSCs. Blue: Hoechst stain. (C) Captured images were converted to 8-bit grayscale and analyzed using ImageJ for vessel length, branching points, segments and nodes. Data are mean ± standard deviation (n = 6, three females and three males). F, female. M, male. One-way ANOVA analysis with Tukey's multiple comparison test. ∗P < 0.05, ∗∗P < 0.01, ∗∗∗P < 0.001, ∗∗∗∗P < 0.0001. ns, not significant.

**Figure 11:**
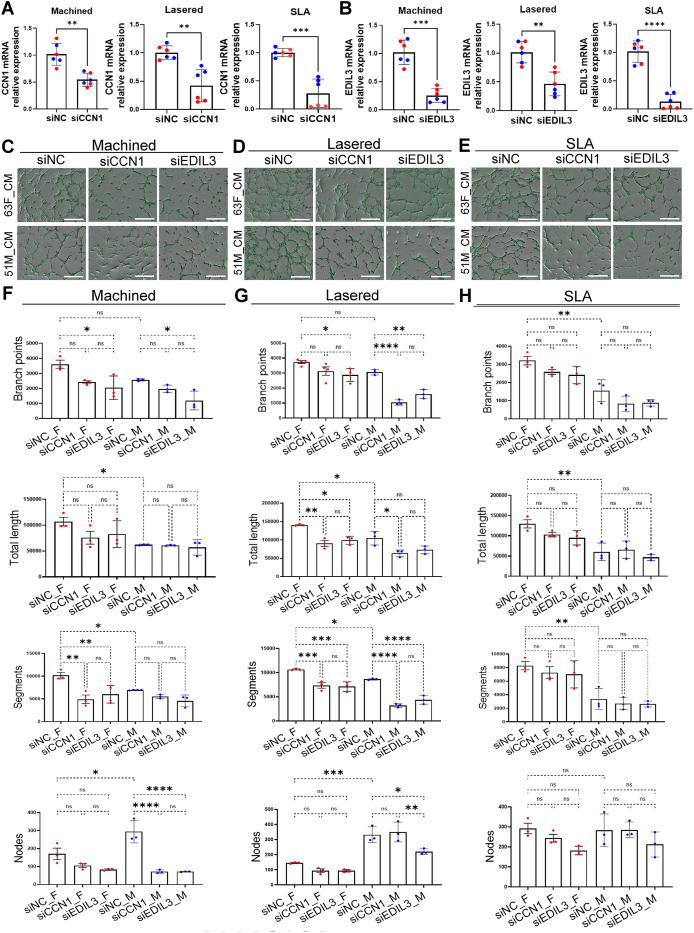
Silencing CCN1 or EDIL3 in gingiva-derived mesenchymal stromal cells (GMSCs) impairs HUVEC angiogenesis. GMSCs were seeded on machined, lasered, or SLA (sand-blasted, large-grit, acid-etched) titanium discs and transfected with siRNAs targeting CCN1 or EDIL3 for 24 h. (A) Relative mRNA expression of CCN1 and EDIL3 after siRNA transfection. NC, negative control siRNA. (B) Representative images of HUVEC tube formation in response to conditioned medium (CM) collected from female (F) and male (M) GMSCs. Images were captured using the ImageXpress Pico system. Scale bars = 500 μm. (C) Quantification of tube formation (branch points, total length, segments, and nodes) using ImageJ. Data are mean ± standard deviation (n = 2, one female and one male). One-way ANOVA analysis with Tukey's multiple comparison test. ∗P < 0.05, ∗∗P < 0.01, ∗∗∗P < 0.001, ∗∗∗∗P < 0.0001. ns, not significant.

To assess how titanium surface modifications affect GMSC-induced angiogenesis, we performed indirect tube formation with HUVECs treated by conditioned medium from GMSCs and direct tube formation assays using coculture of titanium rod with GMSCs and HUVECs. GMSCs derived conditioned medium (GMSCs-CM) was collected 72 h after seeding GMSCs on machined, lasered, or SLA titanium discs. HUVECs cultured in lasered GMSCs-CM showed significantly increased branch points, tube length, segments, and nodes compared to SLA (*P* < 0.05), and both lasered and SLA groups showed more nodes than machined (*P* < 0.01) (Fig. 9). In the direct tube formation model, GMSCs were embedded in GelMA surrounding titanium rods, and HUVECs were added after three days. SEM showed more spindle-shaped cells on lasered rods, while machined and SLA groups had smaller, rounder cells (Fig. 10A). Angiogenesis indicators were significantly higher in the lasered group compared to machined (female, *P* < 0.05; male, *P* < 0.0001) (Fig. 10B and C). Notably, male GMSCs in the lasered group showed greater tube length (*P* < 0.01) and segments (*P* < 0.001) than females. Notably, male lasered group exhibited significantly greater total tube length (*P* < 0.01) and number of segments (*P* < 0.001) compared to the female lasered group. These results suggest that laser-modified titanium enhances GMSC-induced angiogenesis, with males showing stronger early responses. To examine the roles of CCN1 and EDIL3 in GMSC-mediated angiogenesis, we silenced their expression and tested conditioned media on HUVECs (Fig. 11). Across machined, lasered, and SLA titanium surfaces, both siCCN1 and siEDIL3 reduced angiogenic activity (Fig. 11C–H). Taken together, these findings indicate that GMSCs promote HUVEC tube formation through the secretion of CCN1 and EDIL3.

## Discussion

In this study, we demonstrated that laser-modified titanium surfaces stimulate human GMSCs to secrete extracellular vesicles enriched with angiogenic factors, notably CCN1 and EDIL3. Compared to machined and SLA surfaces, laser-modified titanium enhanced GMSC-induced angiogenesis in HUVECs, confirmed by direct and indirect tube formation models. Using a DPSS laser at a speed of 50 mm/s, we created microgrooves (9.84–16.88 μm wide) on titanium.^19^ Prior studies have shown that such microchannels promote osteogenic activity and perpendicular connective tissue attachment.^10^^,^^20^^,^^21^ SEM showed spindle-shaped cells adhering to laser-modified microgrooves, in contrast to the smaller, rounder cells on machined and SLA surfaces, suggesting better peri-implant cell attachment. Besides, lasered titanium significantly upregulated the cytoskeletal gene β-actin in GMSCs compared to machined titanium. Cell morphology correlates with function,^22^ and both CCN1 and EDIL3 regulate integrin-mediated adhesion and cytoskeletal remodeling; these topographical cues may directly influence GMSC behavior.^23^, ^24^, ^25^ Although EDIL3 RNA levels did not significantly increase in the lasered group, both chromatin accessibility at the EDIL3 locus and secreted EDIL3 protein levels were elevated. Furthermore, conditioned media derived from GMSCs after CCN1 or EDIL3 silencing disrupted HUVEC tube formation. Together, these findings suggest that laser-modified microchannels enhance the secretion of CCN1 and EDIL3 by GMSCs, promoting their angiogenic stimulation of HUVECs, which may support improved cell migration and early-stage osseointegration.

We found that GMSCs exhibit sex-dimorphic secretion of CCN1 and EDIL3, along with differences in early angiogenesis in a direct tube formation model. On lasered titanium, male GMSCs secreted more CCN1 and showed greater angiogenic activity—measured by total tube length and segment number—while female GMSCs secreted more EDIL3. Male BMMSCs are known for higher osteogenic potential, whereas female BMMSCs display stronger immunomodulatory effects.^26^ CCN1 regulates both angiogenesis and osteogenesis via integrin pathways, and its higher expression in male GMSCs may enhance early osseointegration.^23^^,^^27^^,^^28^ Nonetheless, clinical data show higher implant failure and marginal bone loss in males,^12^ possibly due to social gender factors such as compliance, oral hygiene, and smoking.^29^ Conversely, EDIL3 promotes inflammation resolution through macrophage efferocytosis, and its increased secretion by female GMSCs may support peri-implant immunomodulation.^30^ To our knowledge, this is the first study to reveal sex-specific differences in GMSC responses to titanium surface modifications, suggesting male GMSCs favor angiogenesis via CCN1, while female GMSCs may support immunomodulation through EDIL3.

GMSCs from supra-crestal connective tissue, share transcriptomic similarities with periodontal ligament stem cells (PDL-MSCs) but have lower osteogenic capacity.^8^^,^^31^ While PDL-MSCs support periodontal and alveolar bone regeneration, GMSCs show strong neuro-regenerative potential, which may aid periodontal regeneration.^14^^,^^32^^,^^33^ Most implant designs focus on intraosseous osseointegration, often overlooking supra-crestal tissue sealing. Histological studies show soft tissue attachment on laser-modified abutments, with favorable long-term outcomes.^10^^,^^34^ In our study, laser-modified titanium surfaces upregulated GMSC genes associated with GO pathways in protein metabolism, communication, proliferation, and migration, supporting active peri-implant soft tissue attachment on lasered microchannels.

Inter-individual variability in primary human GMSCs was a key challenge in this study. Despite this, we observed biologically and clinically relevant effects, particularly in subjects over 40 years of age. Titanium's hardness also hindered histological sectioning by disrupting the soft tissue interface. To address this, we developed a titanium rod-based organoid system that enables real-time cell observation and z-axis analysis via light microscopy—offering a practical alternative to animal models.^35^ In this study, we employed human GMSCs and HUVECs in the organoid model. This study used human GMSCs and HUVECs; future work may incorporate BMMSCs, PDL-MSCs, or immune cells to better mimic the peri-implant microenvironment.

A notable finding in this study was the detection of EV markers TSG101 and flotillin-1 in non-EV fractions after ultrafiltration. While the 100 kDa MWCO filter is widely used for EV isolation, it is limited by low recovery and potential EV membrane disruption under high pressure.^16^^,^^36^ The appearance of these markers in the flow-through may result from ruptured vesicles or soluble forms of exosome-associated proteins. Similar observations have been reported, with TSG101 detected in protein-rich fractions following size-exclusion chromatography.^37^ Thus, <100 kDa permeates may require further clarification, such as ultracentrifugation, before being considered purely non-EV. Importantly, our data show that EVs from the lasered group were enriched in angiogenesis-related proteins CCN1, EDIL3, and flotillin-1, without significant changes in cell lysates or non-EV fractions. Notably, flotillin-1 has also been linked to endothelial activity and can promote angiogenesis.^38^

This is the first study to examine how titanium surface topography affects primary human GMSCs. We found that laser-modified titanium enhances the secretion of extracellular vesicles enriched with angiogenic factors, notably CCN1 and EDIL3. This modification also promoted angiogenesis in HUVECs in both indirect and direct models. These results suggest that laser-modified microchannels provide bioactive cues that stimulate GMSCs to secrete pro-angiogenic factors, potentially supporting early osseointegration.

## Conflicting of interests

The authors declare no conflict of interest.

## References

[1] Brånemark P.I., Adell R., Breine U., Hansson B.O., Lindström J., Ohlsson A. (1969). Intra-osseous anchorage of dental prostheses. I. experimental studies. Scand J Plast Reconstr Surg.

[2] de Campos Kajimoto N., de Paiva Buischi Y., Mohamadzadeh M., Loomer P. (2024). The oral microbiome of peri-implant health and disease: a narrative review. Dent J.

[3] Alam M.N., Ibraheem W., Ramalingam K., Sethuraman S., Basheer S.N., Peeran S.W. (2024). Identification, evaluation, and correction of supracrestal tissue attachment (previously biologic width) violation: a case presentation with literature review. Cureus.

[4] Tsukasaki M., Takayanagi H. (2019). Osteoimmunology: evolving concepts in bone–immune interactions in health and disease. Nat Rev Immunol.

[5] Berglundh T., Lindhe J., Jonsson K., Ericsson I. (1994). The topography of the vascular systems in the periodontal and peri-implant tissues in the dog. J Clin Periodontol.

[6] Atsuta I., Ayukawa Y., Kondo R. (2016). Soft tissue sealing around dental implants based on histological interpretation. J Prosthodont Res.

[7] Larsson L., Pilipchuk S.P., Giannobile W.V., Castilho R.M. (2018). When epigenetics meets bioengineering-a material characteristics and surface topography perspective. J Biomed Mater Res B Appl Biomater.

[8] Kim D., Lee A.E., Xu Q., Zhang Q., Le A.D. (2021). Gingiva-derived mesenchymal stem cells: potential application in tissue engineering and regenerative medicine - a comprehensive review. Front Immunol.

[9] Guarnieri R., Placella R., Testarelli L., Iorio-Siciliano V., Grande M. (2014). Clinical, radiographic, and esthetic evaluation of immediately loaded laser microtextured implants placed into fresh extraction sockets in the anterior maxilla: a 2-year retrospective multicentric study. Implant Dent.

[10] Nevins M., Kim D.M., Jun S.H., Guze K., Schupbach P., Nevins M.L. (2010). Histologic evidence of a connective tissue attachment to laser microgrooved abutments: a canine study. Int J Periodontics Restor Dent.

[11] Olivares-Navarrete R., Hyzy S.L., Chaudhri R.A., Zhao G., Boyan B.D., Schwartz Z. (2010). Sex dependent regulation of osteoblast response to implant surface properties by systemic hormones. Biol Sex Differ.

[12] Chrcanovic B.R., Albrektsson T., Wennerberg A. (2015). Dental implants inserted in male versus female patients: a systematic review and meta-analysis. J Oral Rehabil.

[13] Brizuela-Velasco A., Álvarez-Arenal Á., Pérez-Pevida E. (2021). Logistic regression analysis of the factors involved in the failure of osseointegration and survival of dental implants with an internal connection and machined collar: a 6-year retrospective cohort study. BioMed Res Int.

[14] Zhang Q., Burrell J.C., Zeng J. (2022). Implantation of a nerve protector embedded with human GMSC-derived schwann-like cells accelerates regeneration of crush-injured rat sciatic nerves. Stem Cell Res Ther.

[15] Chu S.F., Huang M.T., Ou K.L. (2016). Enhanced biocompatible and hemocompatible nano/micro porous surface as a biological scaffold for functionalizational and biointegrated implants. J Alloys Compd.

[16] Ono K., Okusha Y., Tran M.T., Umemori K., Eguchi T., Takigawa M. (2023). CCN proteins: methods and protocols.

[17] Carpentier G., Berndt S., Ferratge S. (2020). Angiogenesis analyzer for ImageJ — a comparative morphometric analysis of “endothelial Tube Formation assay” and “Fibrin Bead assay”. Sci Rep.

[18] Lai J.J., Chau Z.L., Chen S.-Y. (2022). Exosome processing and characterization approaches for research and Technology development. Adv Sci.

[19] Lee H.T., Lin C.C. (2019). Enhanced cell proliferation on biomedical titanium surfaces by laser ablation-induced micro- and nanoscale hybrid structures. Mater Trans.

[20] Chen Y.W., Chiang T., Chen I.H. (2024). Titanium surfaces with a laser-produced microchannel structure enhance pre-osteoblast proliferation, maturation, and extracellular mineralization in vitro. Int J Mol Sci.

[21] Teixeira J.F.L., de Souza J.A.C., Magalhães F.A.C. (2023). Laser-modified ti surface improves paracrine osteogenesis by modulating the expression of DKK1 in osteoblasts. J Funct Biomater.

[22] Prasad A., Alizadeh E. (2019). Cell form and function: interpreting and controlling the shape of adherent cells. Trends Biotechnol.

[23] Leu S.J., Lam S.C., Lau L.F. (2002). Pro-angiogenic activities of CYR61 (CCN1) mediated through integrins alphavbeta3 and alpha6beta1 in human umbilical vein endothelial cells. J Biol Chem.

[24] Gasca J., Flores M.L., Jiménez-Guerrero R. (2020). EDIL3 promotes epithelial–mesenchymal transition and paclitaxel resistance through its interaction with integrin αVβ3 in cancer cells. Cell Death Discov.

[25] Heo C.H., Bak S.Y., Kim Y., Ok M.R., Kim S.Y. (2023). Development of an integrin α(v)-based universal marker, capable of both prediction and direction of stem cell fate. Acta Biomater.

[26] Maged G., Abdelsamed M.A., Wang H., Lotfy A. (2024). The potency of mesenchymal stem/stromal cells: does donor sex matter?. Stem Cell Res Ther.

[27] Mao L., Wang L., Xu J., Zou J. (2023). The role of integrin family in bone metabolism and tumor bone metastasis. Cell Death Discov.

[28] Zhao G., Kim E.W., Jiang J. (2020). CCN1/Cyr61 is required in osteoblasts for responsiveness to the anabolic activity of PTH. J Bone Miner Res.

[29] Lipsky M.S., Su S., Crespo C.J., Hung M. (2021). Men and oral health: a review of sex and gender differences. Am J Mens Health.

[30] Kourtzelis I., Li X., Mitroulis I. (2019). DEL-1 promotes macrophage efferocytosis and clearance of inflammation. Nat Immunol.

[31] Srithanyarat S.S., Choosiri M., Sa-Ard-Iam N., Petcharat P., Osathanon T. (2023). Characteristics of mesenchymal stem cells from supracrestal gingival connective tissue. J Periodontol.

[32] Zhao Z., Liu J., Weir M.D. (2022). Periodontal ligament stem cell-based bioactive constructs for bone tissue engineering. Front Bioeng Biotechnol.

[33] Johnston A.P.W., Miller F.D. (2022). The contribution of innervation to tissue repair and regeneration. Cold Spring Harbor Perspect Biol.

[34] Pecora G.E., Ceccarelli R., Bonelli M., Alexander H., Ricci J.L. (2009). Clinical evaluation of laser microtexturing for soft tissue and bone attachment to dental implants. Implant Dent.

[35] Park G., Rim Y.A., Sohn Y., Nam Y., Ju J.H. (2024). Replacing animal testing with stem cell-organoids : advantages and limitations. Stem Cell Rev Rep.

[36] Dilsiz N. (2024). A comprehensive review on recent advances in exosome isolation and characterization: toward clinical applications. Transl Oncol.

[37] Fernández-Rhodes M., Adlou B., Williams S. (2023). Defining the influence of size-exclusion chromatography fraction window and ultrafiltration column choice on extracellular vesicle recovery in a skeletal muscle model. J Exp Biol.

[38] Thalwieser Z., Király N., Fonódi M., Csortos C., Boratkó A. (2019). Protein phosphatase 2A-mediated flotillin-1 dephosphorylation up-regulates endothelial cell migration and angiogenesis regulation. J Biol Chem.

